# Microbial technology with major potentials for the urgent environmental needs of the next decades

**DOI:** 10.1111/1751-7915.12779

**Published:** 2017-08-03

**Authors:** Willy Verstraete, Jo De Vrieze

**Affiliations:** ^1^ Center for Microbial Ecology and Technology (CMET) Ghent University Coupure Links 653 B‐9000 Gent Belgium

## Abstract

Several needs in the context of the water–energy–food nexus will become more prominent in the next decades. It is crucial to delineate these challenges and to find opportunities for innovative microbial technologies in the framework of sustainability and climate change. Here, we focus on four key issues, that is the imbalance in the nitrogen cycle, the diffuse emission of methane, the necessity for carbon capture and the deterioration of freshwater reserves. We suggest a set of microbial technologies to deal with each of these issues, such as (i) the production of microbial protein as food and feed, (ii) the control of methanogenic archaea and better use of methanotrophic consortia, (iii) the avoidance of nitrification and (iv) the upgrading of CO_2_ to microbial bioproducts. The central message is that instead of using crude methods to exploit microorganisms for degradations, the potentials of the microbiomes should be used to create processes and products that fit the demands of the cyclic market economy.

## Introduction

In our current society, we must deal with numerous issues to sustain or even improve the general quality of life of the 7 billion people that currently inhabit the earth. The water–energy–food nexus can be considered one of these key issues for the coming decades (Walker *et al*., 2014). The lingering anticipated climate change adds a fourth factor to this nexus (Beck and Walker, 2013), which makes it more complicated to find suitable solutions that cover multiple aspects.

The development and optimization of environmental microbial technologies over the last century led to multiple applications in this framework. To decrease the pollution from municipalities and industries, the activated sludge process was developed over a century ago (Ardern and Lockett, 1914). Continuous innovation has allowed this process to shift from a mere wastewater clean‐up technology to a provider of renewable energy (Verstraete and Vlaeminck, 2011; De Vrieze *et al*., 2016). Anaerobic digestion of the collected organics hosts a central position in the current wastewater treatment approach, as it serves as the provider of renewable energy. Depending on the process conditions, it also enables the recovery of nutrients and/or organics (Holm‐Nielsen *et al*., 2009; Batstone and Virdis, 2014). Of crucial importance in this context is the evolution of the world energy prices. Currently, energy prices are low, amongst others due to the breakthroughs in the fracking technology, although the long‐term sustainability of this process is highly questionable (Inman, 2014; IEA, 2016). Future energy prices are predicted with a high degree of uncertainty, amongst others due to the major advances in photovoltaic and wind technologies (Cabrera‐Tobar *et al*., 2016).

It appears that for the future wastewater treatment, the production of biogas should no longer be directed to energy recovery, rather biogas and the nutrients present in wastewater must be tuned for reuse by upgrading them into valuable molecules (Cagnetta *et al*., 2016). The recent advances in single cell protein production, which is the production of protein‐rich feed and food, using recovered nutrients and organics, open possibilities for new microbial technological applications to short‐circuit nutrient and energy cycles into more efficient processes (Matassa *et al*., 2015). The combination of wastewater treatment and single cell protein production technologies possesses the potential to substantially influence the water–energy–food–climate nexus of the coming decades.

**Figure 1:**
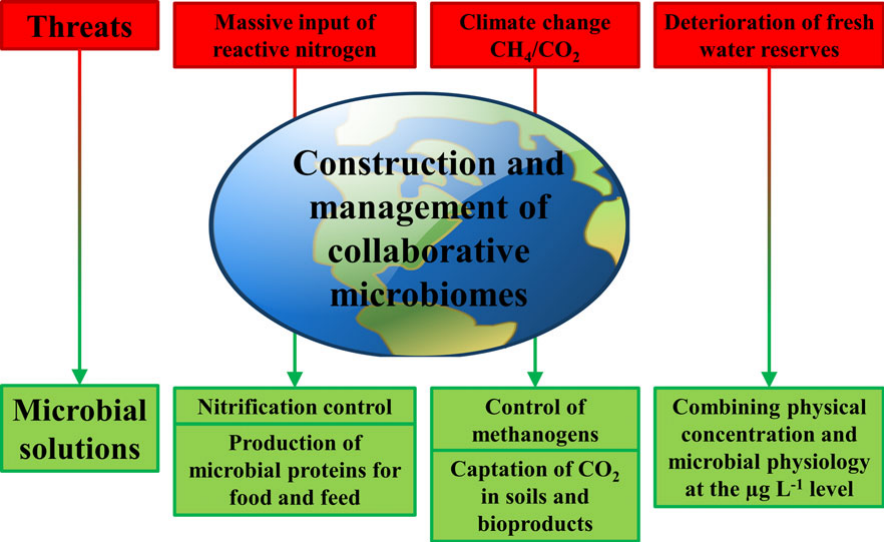
Schematic overview of specific global threats of the coming decades and concomitant potential microbial solutions.

The application of microbial potentialities highlights the emergence of other issues that are directly or indirectly related. We focused on the major challenges of the coming decade, in relation to climate change and sustainability, and the microbial technologies that can be applied to tackle them (Fig. 1).

## The urgent needs

### The planetary boundary conditions: nitrogen is also an issue

The discovery of the Haber–Bosch process for nitrogen fixation in the beginning of the 20th century has been one of the main drivers behind the global population expansion since 1950 (Erisman *et al*., 2008). An important fraction of this reactive nitrogen ends up in the surface waters, which leads to the reactive nitrogen flow strongly surpassing the planetary boundaries, with a high risk of destabilization of the earth ecosystem (Steffen *et al*., 2015). The nitrification process leads to massive losses of reactive nitrogen in various natural and manmade ecosystems, caused by both washout from the soil and denitrification to N_2_ (Subbarao *et al*., 2006). Time has come to focus on controlling nitrification, for instance by implementing a new generation of slow‐release fertilizers and, if possible, plant‐generated nitrification inhibitors (O'Sullivan *et al*., 2016). A similar approach should be applied in wastewater treatment in which nitrogen recovery, rather than dissipation *via* the nitrification/denitrification process should be targeted. Nitrification should be labelled as an environmentally undesirable process, and major efforts should be undertaken to avoid it. The need to recover/reuse the nitrogen present in our secondary resources does not originate from a source deficiency, unlike certain rare earth elements (Hennebel *et al*., 2015), but from a sink overload. Dealing in a drastically different way with nitrogen relates with the optimal use of fossil energy and preservation of the environmental quality.

At present, 1–2% of the energy consumption on earth is used to fuel the Haber–Bosch process for nitrogen fixation. Yet, depending on the human diet (vegetarian or carnivorous), only 4–16% of this nitrogen is consumed (Galloway and Cowling, 2002; Matassa *et al*., 2015). To sustain the food (nitrogen) supply to the growing world population, soy production has boomed over the last two decades (Dalin *et al*., 2012), with an increase from 17 million tons in 1960 to 230 million tons in 2008 (Hartman *et al*., 2011). The growing risk of irreversible deforestation and the fact that the production of soya bean coincides with 0.3–0.6 kg CO_2_ equivalents per kg of fresh weight do not support a sustainable expansion of this potential and imply the need for alternative nitrogen sources to be used as feed or food (Dalin *et al*., 2012; Castanheira and Freire, 2013). An alternative solution lies in the production of single cell proteins, also called microbial proteins. These do not compete for land usage with other crops, and much higher nitrogen efficiencies can be obtained, especially when used directly as food source (Matassa *et al*., 2015; Pikaar *et al*., 2017). The key aspect of microbial protein production for feed and food lies in the ability to use recovered nitrogen from different sources, such as source‐separated urine (Maurer *et al*., 2003; Luther *et al*., 2015) and the liquid fraction of digestate (Desloover *et al*., 2012). This has multiple advantages, as it (i) avoids additional costs for reactive nitrogen conversion to nitrogen gas, (ii) decreases the amount of nitrogen to be fixed by Haber‐Bosch and (iii) prevents excessive accumulation of reactive nitrogen in the biosphere.

### Climate change: the diffuse methane emissions and their abatement

Anaerobic digestion provides the possibility to stabilize organic waste streams, which results in the controlled production of CH_4_‐rich biogas. This biogas can be (i) used for flexible electricity and heat production (Szarka *et al*., 2013), (ii) upgraded to biomethane for injection on the smart gas grid (Lund *et al*., 2012; Junne and Kabisch, 2017) or (iii) used as carbon source for microbial feed and food production (Strong *et al*., 2015). Part of the methane, however, remains dissolved in the effluent or digestate (Cakir and Stenstrom, 2005; Liebetrau *et al*., 2010). For low‐strength wastewaters, this can easily exceed 25% (Hartley and Lant, 2006), which reflects a simultaneous high loss in energy recovery potential and emission of a potent greenhouse gas to the atmosphere, as it has a global warming potential of 28 CO_2_ equivalents (Saunois *et al*., 2016). Other engineered or natural anaerobic microbial ecosystems, such as septic tanks, manure pits, landfills, sewers, activated sludge, rice paddies, wetlands and particularly the methanogens active in ruminants also contribute to the diffuse release of methane (Johnson *et al*., 2007; Perez‐Barberia, 2017). The diversity of sources of diffuse methane emissions implies the need for case‐specific solutions.

The dissolved methane, often at supersaturation (Hartley and Lant, 2006), in the liquid effluent of anaerobic digesters can be mitigated, for example, *via* the methalgae approach (van der Ha *et al*., 2011). Methanogenesis in the rumen can be inhibited by adding, for example, 3‐nitrooxypropanol to the feed (Romero‐Perez *et al*., 2014; Duin *et al*., 2016) or by applying antibodies to prevent adhesion of *Methanobrevibacter* (Ng *et al*., 2016). However, methanogenesis might be essential for cellulose digestion in the rumen (Mason and Stuckey, 2016), which accentuates the need to look for other routes than ruminants to supply high‐quality proteinaceous foods. Methane emission from wetlands or rice paddies can be decreased by short‐term drainage or temporary oxygen supply (Ratering and Conrad, 1998; Arends *et al*., 2014). Methane emissions from lakes can be controlled by steering aquatic trophic interactions to minimize grazing of methanotrophs by zooplankton (Devlin *et al*., 2015). This demonstrates that there is huge potential of challenging microbial engineering systems to deal with diffuse methane emissions and their impact on climate change.

### Microbial biotechnology for CO_2_ capture

The atmospheric CO_2_ concentration has been increasing since the mid‐18th century in response to human activities (Menon *et al*., 2007). To reduce global CO_2_ emissions to 80% of the levels of 1990 by 2050, we need to achieve a 4200 Mt CO_2_ sequestration per year (Mac Dowell *et al*., 2017). To deal with this challenge, we rely on carbon capture and storage (CCS) and carbon capture and utilization (CCU). The overall perspective of the chemical conversion of CO_2_ to organic compounds, such as urea and methanol, is estimated to contribute only 1% to the necessary CO_2_ reduction requirement (Mac Dowell *et al*., 2017). This emphasizes the need to consider alternative options, amongst which microbial processes to contribute to CO_2_ sequestration deserve to be explored. The soil microbial ecosystem, as well as the ocean ecosystem, has been important sinks for CO_2_, which partially mitigated the human impact on the carbon cycle (Menon *et al*., 2007). The importance of the soil microbial community in the sequestration of carbon suggests that there is a huge potential to maximize CO_2_ sequestration through microbial community engineering.

A change in land use from arable land to grassland entails an average 18% higher carbon sequestration, which relates with a yearly carbon input of 0.75 tonnes C ha^−1^ year^−1^ (Kampf *et al*., 2016). A soil with a limited degree of manipulation reaches a higher degree of microbial homoeostasis (Cleveland and Liptzin, 2007), which allows a more efficient carbon sequestration. This type of soil management can be of great value in the context of the anticipated climate change (Fontaine and Barot, 2005; Manzoni *et al*., 2012). An alternative approach lies in the addition of charcoal or biochar to the soil, which (i) is a direct addition of long‐term stable carbon to the soil, (ii) improves the overall soil quality and (iii) can adsorb nutrients to increase their plant bioavailability (Lehmann *et al*., 2006; Laird, 2008; Prost *et al*., 2013).

The concept of carbon sequestration can also be approached by using concentrated CO_2_ sources. Microbial electrosynthesis allows the generation of valuable products from electricity, using CO_2_ or other organic feedstocks as carbon source (Nevin *et al*., 2010; Rabaey and Rozendal, 2010; Lovley, 2011). This enables the production of acetate (Gildemyn *et al*., 2015), butyrate (Ganigue *et al*., 2015) and other commodity chemicals (Nevin *et al*., 2011; Arends *et al*., 2017). These chemicals, when subjected to the process of microbial chain elongation, can be converted to medium chain fatty acids with a higher economic value, such as caproate and caprylate, which serve as bio‐based building blocks for the chemical industry (Agler *et al*., 2012; Spirito *et al*., 2014; Angenent *et al*., 2016). A key point in this process remains the energy‐efficient harvesting of these chemicals to obtain a concentrated stream with high product quality (Agler *et al*., 2011; Gildemyn *et al*., 2015; Andersen *et al*., 2016).

### The ongoing deterioration of freshwater reserves

The intensive usage of nutrients, mainly N and P, results in at least a fraction of these nutrients ending up in the natural water bodies, which may result in eutrophication (Conley *et al*., 2009; Coppens *et al*., 2016). The anticipated increase in the human population is predicted to result in further eutrophication of terrestrial, freshwater and nearshore marine ecosystems (Tilman *et al*., 2001). Micropollutants are often insufficiently removed and accumulate in increasing amounts and even more staggering diversity in the surface waters (Margot *et al*., 2015). Although through river bank filtration > 90% of micropollutants can be removed in a period of 4 years, certain micropollutants show persistent behaviour, which may have a long‐term impact on the surface water biological quality (Hamann *et al*., 2016). This also directly affects our drinking water reserves (ground‐ and surface water), although drinking water production technologies are adequate to remove most micropollutants (Luo *et al*., 2014). However, this does not necessary result in a prompt change in suitable regulations (Sedlak, 2016). The main issue lies in the extent of removal, implying that future technologies need to deal with concentrations down to 1 μg l^−1^. These can be a combination of physical processes, for example increasing the concentrations by membrane technologies, and the use of the versatile bioconversion potential of naturally selected microbiomes.

The potential impact of persistent micropollutants implies the need for end‐of‐pipe treatment technologies in wastewater treatment plants to safeguard the aquatic ecosystems. Biological treatment can be a suitable approach to remove micropollutants from drinking water, but often requires bioaugmentation of suitable strains to be effective (Benner *et al*., 2013). A potential opportunity is the application of ammonium oxidizing bacteria that can co‐metabolize certain micropollutants down to the μg l^−1^ level (Kassotaki *et al*., 2016). Methane‐oxidizing communities can also be used for this purpose (Benner *et al*., 2015). Technologies combining physical concentration and advanced microbial physiology to deal with the enormous diversity of micropollutants still have a long way to go to safeguard our drinking and process water quality.

## Cooperating with microorganisms

The crucial role of microorganisms in tackling the abovementioned challenges of the coming decades is apparent. To engage in more energy‐efficient processes with higher removal rates and efficiencies, we need to be able to control the microorganisms *via* an integrated approach. The way to go is to consider the microbial community as a structured three‐dimensionally organized entity, as used in the microbial resource management (MRM) (Marzorati *et al*., 2008; Read *et al*., 2011) and mixed culture biotechnology (Kleerebezem and van Loosdrecht, 2007) approaches. This also counts for the fields of human health and food safety, as total elimination of evolving microbial communities, rather than collaborating with them, leads to multiple side issues, such as antibiotic resistance (Baquero *et al*., 2008; Berendonk *et al*., 2015). An excellent example to avoid the usage of antibiotics is the biofloc technology as applied in aquaculture (Crab *et al*., 2012). Overall, a better understanding of the basics of the microbial communities in key processes will be crucial to tackle a set of important environmental challenges in the coming decades.

## Conflict of interest

None declared.
